# Metabolic Signatures of Gestational Weight Gain and Postpartum Weight Loss in a Lifestyle Intervention Study of Overweight and Obese Women

**DOI:** 10.3390/metabo10120498

**Published:** 2020-12-04

**Authors:** Chung-Ho E. Lau, Victoria Taylor-Bateman, Panagiotis A. Vorkas, Gonçalo Graça, Thanh-Huyen T. Vu, Lifang Hou, Elena Chekmeneva, Timothy M. D. Ebbels, Queenie Chan, Linda Van Horn, Elaine Holmes

**Affiliations:** 1 Section of Nutrition, Department of Metabolism, Digestion and Reproduction, Imperial College London, London SW7 2AZ, UK; v.j.taylor-bateman@qmul.ac.uk; 2 Department of Epidemiology and Biostatistics, School of Public Health, Imperial College London, London W2 1PG, UK; q.chan@imperial.ac.uk; 3 William Harvey Research Institute, Barts and The London School of Medicine and Dentistry, Queen Mary University of London, London EC1M 6BQ, UK; 4 Section of Biomolecular Medicine, Department of Metabolism, Digestion and Reproduction, Imperial College London, London SW7 2AZ, UK; p.vorkas@imperial.ac.uk; 5 Institute of Applied Biosciences, Centre for Research and Technology Hellas, 57001 Thessaloniki, Greece; 6 Section of Bioinformatics, Department of Metabolism, Digestion and Reproduction, Imperial College London, London SW7 2AZ, UK; g.gomes-da-graca@imperial.ac.uk (G.G.); t.ebbels@imperial.ac.uk (T.M.D.E.); 7 Department of Preventive Medicine, Feinberg School of Medicine, Northwestern University, Chicago, IL 60611, USA; huyenvu@northwestern.edu (T.-H.T.V.); l-hou@northwestern.edu (L.H.); lvanhorn@northwestern.edu (L.V.H.); 8 National Phenome Centre and Section of Bioanalytical Chemistry, Department of Metabolism, Digestion and Reproduction, Imperial College London, Hammersmith Campus, IRDB Building, London W12 0NN, UK; e.chekmeneva@imperial.ac.uk; 9 MRC Centre for Environment and Health, Imperial College London, London W2 1PG, UK; 10 Centre for Computational and Systems Medicine, Health Futures Institute, Murdoch University, Perth, WA 6150, Australia

**Keywords:** metabolic phenotyping, metabolomics, blood lipids, LC-MS, NMR, gestational weight gain, lifestyle intervention

## Abstract

Background: Overweight and obesity amongst women of reproductive age are increasingly common in developed economies and are shown to adversely affect birth outcomes and both childhood and adulthood health risks in the offspring. Metabolic profiling in conditions of overweight and obesity in pregnancy could potentially be applied to elucidate the molecular basis of the adverse effects of gestational weight gain (GWG) and postpartum weight loss (WL) on future risks for cardiovascular disease (CVD) and other chronic diseases. Methods: Biofluid samples were collected from 114 ethnically diverse pregnant women with body mass index (BMI) between 25 and 40 kg/m^2^ from Chicago (US), as part of a randomized lifestyle intervention trial (Maternal Offspring Metabolics: Family Intervention Trial; NCT01631747). At 15 weeks, 35 weeks of gestation, and at 1 year postpartum, the blood plasma lipidome and metabolic profile of urine samples were analyzed by liquid chromatography mass spectrometry (LC-MS) and ^1^H nuclear magnetic resonance spectroscopy (^1^H NMR) respectively. Results: Urinary 4-deoxyerythronic acid and 4-deoxythreonic acid were found to be positively correlated to BMI. Seventeen plasma lipids were found to be associated with GWG and 16 lipids were found to be associated with WL, which included phosphatidylinositols (PI), phosphatidylcholines (PC), lysophospholipids (lyso-), sphingomyelins (SM) and ether phosphatidylcholine (PC-O). Three phospholipids found to be positively associated with GWG all contained palmitate side-chains, and amongst the 14 lipids that were negatively associated with GWG, seven were PC-O. Six of eight lipids found to be negatively associated with WL contained an 18:2 fatty acid side-chain. Conclusions: Maternal obesity was associated with characteristic urine and plasma metabolic phenotypes, and phospholipid profile was found to be associated with both GWG and postpartum WL in metabolically healthy pregnant women with overweight/obesity. Postpartum WL may be linked to the reduction in the intake of linoleic acid/conjugated linoleic acid food sources in our study population.

## 1. Introduction

Overweight and obesity are cardinal features of metabolic syndrome, and are triggers for further onset of hypertension, dyslipidemia, insulin resistance and other factors that are components of metabolic syndrome [1]. Additionally, maternal health is associated with both acute and long-term impacts of the health of offspring including the development of metabolic syndrome in early childhood [2,3,4]. Obesity amongst women of reproductive age is increasingly common in developed countries. In the US, the majority of women of reproductive age are overweight or obese [5,6]. Maternal obesity is linked to gestational diabetes, stillbirth and caesarean delivery [7], and also adversely affects childhood weight gain trajectories, raising the risk for developing future cardiometabolic, respiratory and cognitive-related health outcomes in offspring [8,9,10]. These health outcomes are further worsened among women with excessive gestational weight gain (GWG), associated with postpartum weight retention [11] and it has been shown that their children are 30–45% more likely to become obese both in childhood and adulthood [12]. Women tend to be conscious of providing a healthy start to life for their offspring and thus are often motivated to adopt diet and lifestyle changes, thereby providing a potential window of opportunity for intervention [13]. Recent lifestyle intervention research programs such as the UPBEAT, GLOW, GeliS, and the LIFE-Moms studies were funded to promote the aim of reducing the heath burden of excess GWG in pregnant women [14,15,16,17]. The Maternal-Offspring Metabolics: Family Intervention Trial (MOMFIT) is one of seven different randomized clinical trials aimed at restriction of excess GWG funded by NIDDK/NHLBI LIFE-Moms Consortium [16]. Pregnant women with a body mass index (BMI) of 25–40 kg/m^2^ were randomized to either a lifestyle intervention involving a DASH (Dietary Approaches to Stopping Hypertension)—type diet especially suited to pregnant women (called “Mama DASH”) that was calorie adjusted to meet nutrient needs for each woman or a usual-care control group [18]. Self-monitoring, moderate physical activity and adequate sleep were recommended and reinforced through weekly coaching calls and group sessions mostly conducted via webinars for participants assigned to the intervention group.

Metabolic profiling is a robust tool for advancing knowledge of the consequences of maternal obesity at the molecular level. We, and others, have shown that obesity and related co-morbidities, including cardiovascular disease have a profound impact on the metabolome (the total chemical composition of all low molecular weight compounds in a biological fluid or tissue) [19,20,21,22,23]. Metabolic profiling has also been applied to document the series of metabolic changes throughout the course of pregnancy and pregnant women have a distinct metabolic profile compared with non-pregnant women [24]. Moreover, metabolic profiling is an established technique for identifying biomarkers of health risks in mother-child cohorts addressing issues such as gestational diabetes, preterm birth, and fetal growth restriction [25,26,27]. For example, women with normal weight have different placenta metabolic profiles compared with obese women [28], and the maternal metabolome during pregnancy has been associated with newborn body fat [29].

In this study, we applied a systems approach based on nuclear magnetic resonance spectroscopy and mass spectrometry profiling to characterize the metabolic changes related to reduced GWG and greater weight-loss postpartum (WL) in pregnant women enrolled in diet and lifestyle intervention studies. Specifically, we analyzed urine and blood plasma samples collected as part of the MOMFIT trial using a combination of, ^1^H nuclear magnetic resonance spectroscopy (^1^H NMR) to analyze urine samples, and reverse phase ultra-performance liquid chromatography-time of flight-mass spectrometry (RP-UPLC-qTOF-MS) to generate lipid profiles from the plasma samples. In this study, we identified metabolites that were influenced by maternal BMI, GWG and postpartum WL.

## 2. Results

### 2.1. ^1^H NMR Detectable Urine Metabolome Correlates with BMI in Our Study Cohort

**Figure 1:**
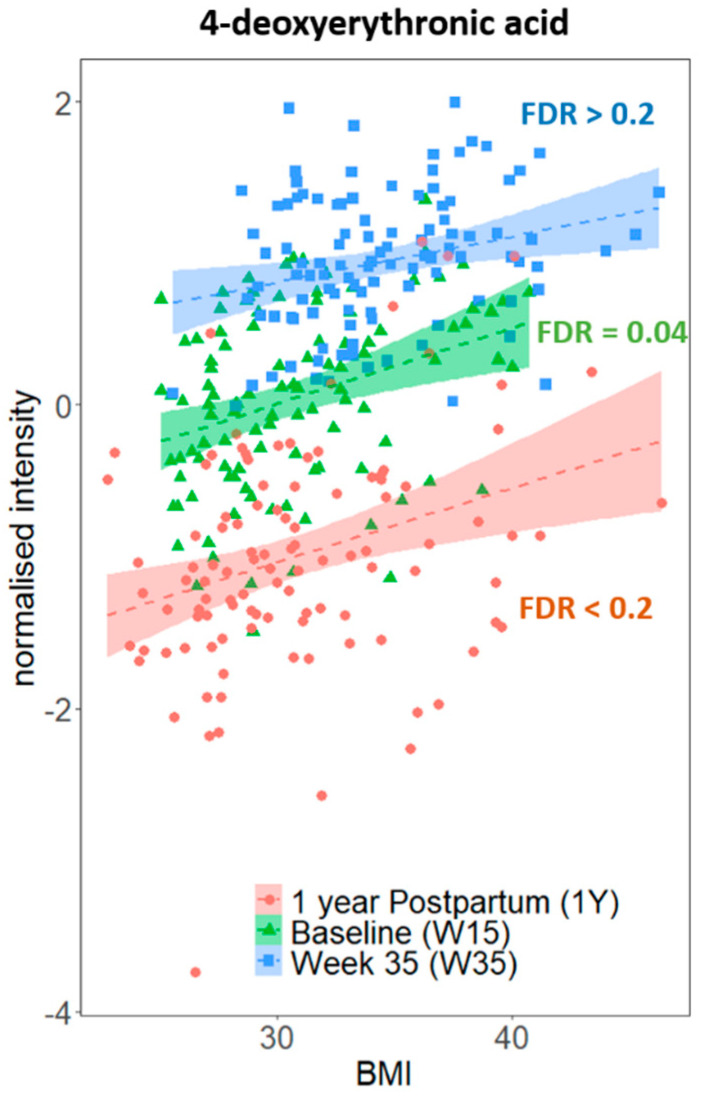
Relationship between urinary 4-deoxyerythronic acid and BMI at three different sampling timepoints. Signal integrals were normalized to median-fold change and log-transformed, and Pearson’s correlation *p*-values before multiple testing correction are shown. FDR, false discovery rate.

As stages of pregnancy were associated with urinary metabolic variations in our study samples (Supplementary Figure S1), we have stratified the data analysis by study sampling timepoints—Baseline (W15), Week 35 (W35), and at 1 year Postpartum (1Y). We aimed to identify metabolites associated with measures of obesity in our study cohort, rather than with pregnancy stage. Univariate Pearson’s correlation analyses were performed on each of the aligned ^1^H NMR spectral peak signals against BMI separately for each timepoint, and 4-deoxyerythronic acid (*r* = 0.33, false discovery rate (FDR) = 0.04) and 4-deoxythreonic acid (*r* = 0.36, FDR = 0.02) were identified as positively correlated to baseline BMI after multiple testing correction. No signals were found significantly correlated with W35 or 1Y BMI after multiple testing corrections, although positive (non-significant) trends between 4-deoxyerythronic acid and BMI remained evident at both timepoints (Figure 1). There was no evidence of correlation between either 4-deoxyerythronic acid or 4-deoxythreonic acid, or any other metabolite and GWG or WL post-delivery.

### 2.2. Alteration in Plasma Phospholipid Levels Are Strongly Associated with GWG and WL

**Figure 2:**
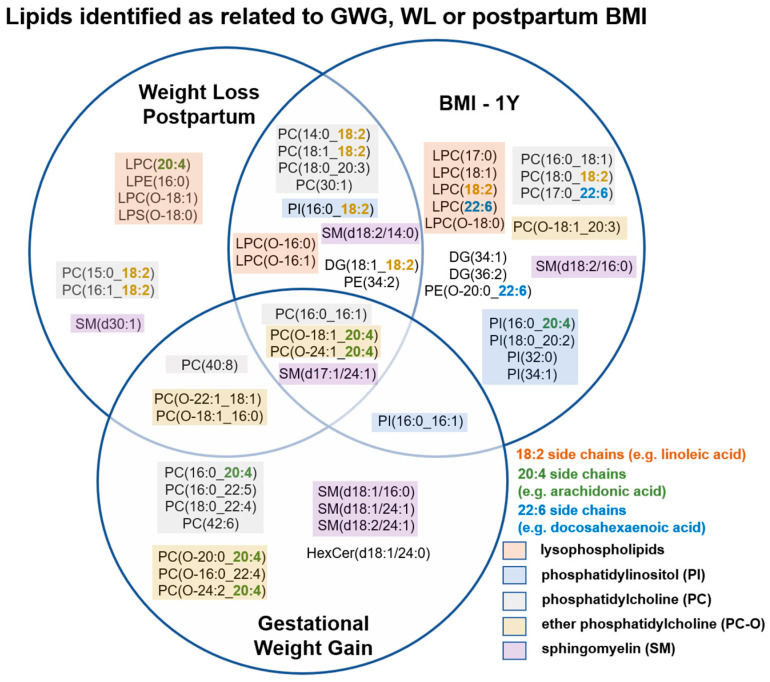
The Venn diagram shows blood lipids associated to GWG, WL or 1Y BMI in the study. Lipids significantly associated to either GWG, WL or 1Y BMI were identified through correlation analyses. Pearson’s correlation analyses were performed on the 1127 MS positive ion mode features that passed quality control, and features of FDR < 0.05 were considered significantly correlating to gestational weight gain, postpartum weight loss or BMI at 1 year postpartum. Many of the lipids related to GWG, WL, or postpartum BMI were lysophospholipids, phosphatidylinositols, phosphatidylcholines, ether phosphatidylcholines, or sphingomyelins, and many of the polyunsaturated lipids annotated contain 18:2, 20:4 or 22:6 side chains. Key: PC, phosphatidylcholine; PI, phosphatidylinositol; PE, phosphatidylethanolamine; SM, sphingomyelin; LPC, lyso phosphatidylcholine; LPE, lysophosphatidylethanolamine; LPS, lysophosphatidylserine; DG, diacylglycerol; HexCer, hexosylceramide. Note, our analysis did not discriminate between allyl-(plasmanyl) or alkenyl (plasmenyl) linked ether lipids which differ only in the position of the C=C double bond in the sn-1 position and produce the same fragment ions. These were labelled as either PC-O or LPC-O or LPS-O.

A total of 1127 positive mode ions in the RP-UPLC-QTOF analysis of blood plasma lipid untargeted profiles passed quality control criteria and were selected for further data analysis. The effect of pregnancy status on the lipidome was prominent, and clear clustering by sampling time-point could be observed in the 1st component of the principal component model (Supplementary Figure S2). Pearson’s correlation was performed to identify features significantly correlated to either GWG, WL or 1Y BMI (FDR < 0.05), which were then further annotated using MS/MS data collected during the run. A total of 57 lipids that were associated with at least one of the outcome measures were annotated (Supplementary Table S1). These mainly consisted of phospholipids including multiple lysophospholipids, phosphatidylinositols (PI), phosphatidylcholines (PC), ether phosphatidylcholines (PC-O) and sphingomyelins (SM) (Figure 2).

**Figure 3:**
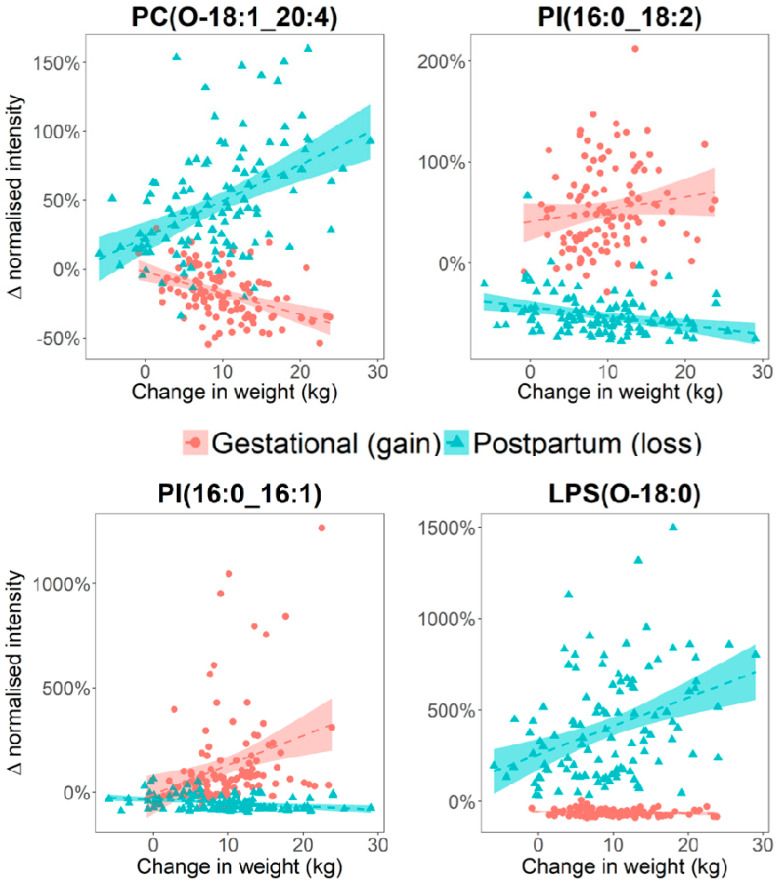
Scatter plots illustrating opposite direction of associations are observed between metabolite level, and GWG or WL. The Y-axes represent the change in metabolite intensity between the Baseline and WK35 (for GWG) or between the 1Y and W35 (for WL) samples, and these were calculated per-participant and expressed in percentage change.

We also noted these included a significant number of 18:2 (11/57), 20:4 (7/57), and 22:6 (3/57) side chain containing lipids. Linear regression analysis was performed in order to relate GWG and WL to plasma lipid percentage changes in the corresponding sampling periods. Seventeen lipid species were found to be associated with GWG and 16 lipids were associated with WL (Table 1). Three phospholipids—PI (16:0_16:1), PC (16:0_20:4), and PC (16:0_22:5) were positively associated with GWG, all containing a 16:0 side chain. Amongst the 14 lipids found to be negatively associated with GWG, seven were ether phosphatidylcholines (PC (O-18:1_20:4), PC (O-20:0_20:4), PC (O-18:1_16:0), PC (O-24:2_20:4), PC (O-22:1_18:1), PC (O-16:0_22:4), and PC (O-24:1_20:4)); three were sphingomyelins—SM (d17:1/24:1), SM (d18:1/24:1), and SM (d18:2/24:1) all containing a 24:1 side chain; two were polyunsaturated PC—PC (42:6) and PC (40:8); and 2 were lysoPC-O—LPC (O-16:1) and LPC (O-18:1). Eight lipids were positively associated with WL; these included three lysoPC-O LPC (O-16:0), LPC (O-16:1), LPC (O-18:1), two PC-O PC (O-18:1_20:4) and PC (O-22:1_18:1), and PC (40:8), LPS (O-18:0), and SM (d17:1/24:1). Notably, 6 out of eight lipids found negatively associated with WL contained an 18:2 fatty acid side chain—PC (18:1_18:2), PC (16:1_18:2), PC (14:0_18:2), PC (15:0_18:2), PI (16:0_18:2), and DG(18:1_18:2). Of the 14 lipids associated with GWG, seven were also associated with WL but with their direction of association reversed (Table 1, Figure 3). Some lipids such as LPS(O-18:0), were more strongly associated with WL than GWG (Figure 3).

Additionally, we noted that metabolite levels of four phosphatidylinositols PI (16:0_20:4), PI (18:0_20:2), PI (32:0) and PI (34:1) were positively correlated with postpartum BMI, and five lysophospholipids—LPC (17:0), LPC (18:1), LPC (18:2), LPC (22:6) and LPC (O-18:0) as well as two other lipids with 22:6 side chains (docosahexaenoic acid)—PC (17:0_22:6) and PE(O-20:0_22:6) were negatively correlated with postpartum BMI. These lipids were not directly associated with GWG or WL (Figure 3).

### 2.3. The Impact of Lifestyle Intervention on Plasma Lipid Levels

**Figure 4:**
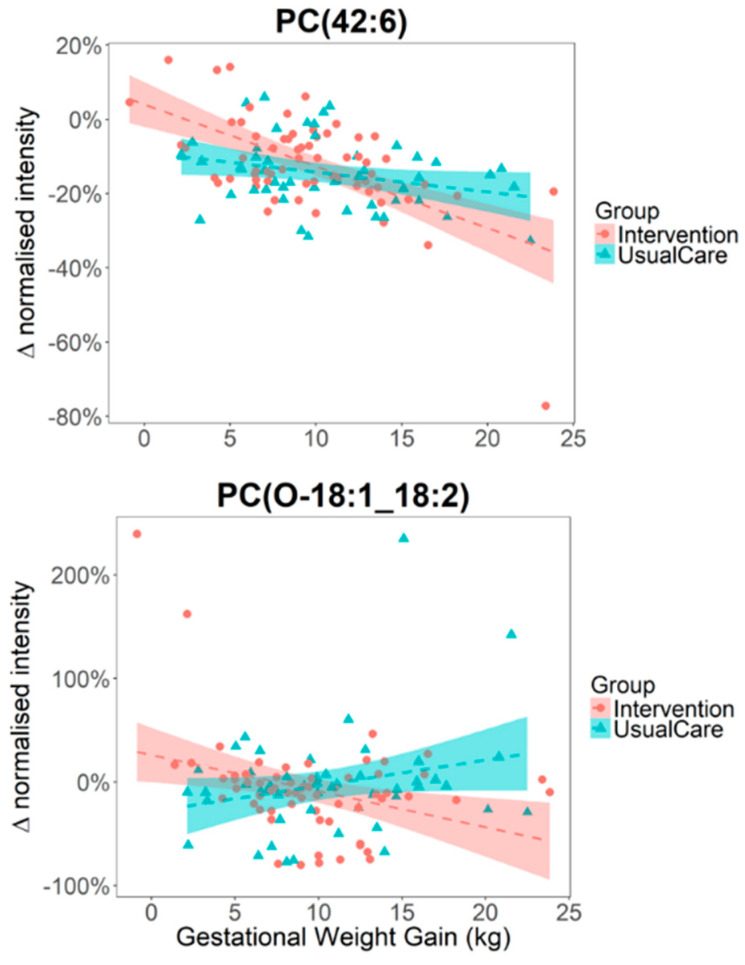
Intervention resulting in modest interactions to metabolite-GWG associations. The y-axes represent the change in metabolite intensity between the Baseline and W35 samples calculated per-participant and expressed in percentage change.

The study participants were split into two groups, with one group assigned to “usual care” and the other as “intervention”, who were prescribed DASH diet-specific calorie goals to follow during the 2nd and 3rd trimesters of their pregnancies along with coaching calls and further monitoring. The effects of intervention on the urine and plasma metabolite levels at W35 and 1Y were not significantly different between the “usual care” and “intervention” groups as assessed by Student’s *t*-test. We next considered if the intervention modulated the associations between lipid level and GWG, and tested each of the 57 annotated lipids with ANOVA (linear model) by including an interaction term between GWG and study intervention grouping in the model. We found that metabolite-GWG associations of four lipids—PC (O-18:1_18:2), PC (18:1_18:2), PC (18:0_20:3) and PC (42:6) were potentially modulated by the effect of study intervention (nominal *p* < 0.05, 0.05 < FDR < 0.2), however none of them reached significance after accounting for multiple testing (Figure 4).

## 3. Discussion

This study identified urine and plasma lipid molecular markers of maternal obesity in a cohort of healthy pregnant women with overweight/obesity who were randomized as part of a diet and lifestyle intervention trial. Whilst previous metabolic profiling studies have examined metabolites associated with BMI or GWG during pregnancy [30], there is currently a lack of published information on the metabolomic signature of postpartum WL—a parameter of considerable interest to postnatal women participating in lifestyle intervention studies. Several of the phospholipids associated with GWG were also found to be associated with post-partum WL, but in the reversed direction, suggesting that the metabolic pathways associated with WL likely largely substantially overlap with those associated with GWG.

Specifically, the current study found that all three phospholipids observed to be positively associated with GWG contained a 16:0 fatty acid (palmitate) side chain. Palmitate is the primary product of *de novo* lipogenesis, and is enhanced during pregnancy [31]. A study by Postle et al. showed that the increased plasma phosphocholine levels throughout pregnancy was dominated by individual lipids containing palmitate (16:0) rather than stearate (18:0) at the *sn-1* position, and suggested that adapted maternal hepatic PC metabolism served to provide the fetus with an adequate supply of 22:6(n3) [32]. Elevated palmitate concentrations in both maternal and placental blood have previously been associated with both BMI and gestational diabetes [33].

Ether phosphatidylcholines (PC-O) made up a large proportion of the lipids that were negatively associated with GWG in our analysis, a previous study has shown that maternal level of ether phosphatidylcholines (PC-O) containing fatty acid 20:4 (e.g., arachidonic acid) were negatively associated with newborn body fat percentage, and may exert a protective effect against obesity development [34]. Notably, six of the eight lipids that were negatively associated with WL contained an 18:2 side chain. Since linoleic acid/ conjugated linoleic acid are diet-derived, and are major isomers for 18:2 fatty acid, our analysis suggests WL maybe linked to the reduction in the intake of linoleic acid/conjugated linoleic acid food source in the study sample. Nuts, seeds, meats and eggs are all dietary sources of linoleic acid, and preliminary analyses from our study have shown that meat and legume intakes were correlated with levels of a subset of phospholipids containing an 18:2 sidechain at 1-year postpartum (data not shown). Linoleic acid is a major natural ligand for cell signaling biomolecules peroxisome proliferator-activated receptors (PPARs) [35], which regulate lipid and glucose metabolism. The level of linoleic acid detected in human adipose tissue has steadily increased over the last 50 years [36]. Four of the eight lipids directly associated with WL in our study were lysophospholipids, previously reported to be associated with WL in children [37], and were inversely associated with risk of development type 2 diabetes [38]. Additionally, we noted several phosphatidylinositols (PI) were positively associated with postpartum BMI; PI are important building blocks for cell signaling molecules in the insulin signaling pathway which also regulate diabetes, obesity and cell growth [39]. Phospholipids with 22:6 side chains (docosahexaenoic acid) were found negatively associated with postpartum BMI, but not associated with either GWG or postpartum WL; docosahexaenoic acid supplements taken by pregnant/lactating mothers have previously been shown to lower BMI [40].

Strengths of this study include the focus on diet assessment and intervention among these pregnant women with overweight or obesity, offering more specific exploration of the possible metabolomic associations. The major limitation is the relatively small sample size and its consequences. In both the urine ^1^H NMR and the plasma lipid datasets, the principal component analysis (PCA) scores plots show that there were dominant shifts in metabolite profiles between the first and third trimester, with profiles reversed at 1 year postpartum (Supplementary Figure S1). The relatively low variance explained by the first two principal components, particularly for the urine dataset (13.01%), further highlights the challenge of identifying weight-related changes against the extreme metabolic shifts caused by pregnancy, and other genetic and environmental factors. Although we did not observe significant associations of any metabolites with either GWG or WL in our urinary ^1^H NMR profile—possibly due to the large within-individual temporal and biological variabilities that are characteristic of the urinary metabolome [41], we did however identify urinary 4-deoxyerythronic acid and 4-deoxythreonic acid as positively associated with BMI in our study population. Levels of urinary 4-deoxyerythronic acid were found to be higher during pregnancy (Figure 1) and were highest in the 3rd trimester, which corroborates previously published research [42]. Additionally, we observed 4-deoxyerythronic acid and 4-deoxythreonic acid to be well correlated during pregnancy, particularly at the late stage of pregnancy (WK15, Pearson’s r = 0.31, nominal *p* = 0.001; WK35, Pearson’s *r* = 0.68, nominal *p* = 4 × 10^−16^), but not in the postpartum urine samples (Pearson’s *r* = 0.09, *p* = 0.34). The differential correlation during pregnancy may reflect a biological role for both isomers during pregnancy, or the stronger correlation during late pregnancy may be the consequence of the increased production of one or the other compound. Since 4-deoxythreonic acid has been reported to be increased in sleep deprivation [43] and major depressive disorder [44], it would appear unlikely that the formation of one of these isomers is a result of a spontaneous chemical reaction. Recently, urinary 4-deoxyerythronic acid was shown to be associated with BMI in healthy children [45] and it is possible that 4-deoxyerythronic acid—a catabolite of threonine metabolism, may play an important role in development during early-life [46]. Future maternal and children lifestyle/dietary intervention studies could consider targeting metabolites within the threonine metabolic pathway.

One of the key exploratory objectives of the MOMFIT study was to identify molecular markers of response to the lifestyle intervention as prescribed in the study trial during the pregnancy period. One previous study reported reduced levels of sphingomyelins were associated with lifestyle intervention in obese children [47], and in a recent UK based lifestyle intervention study of 1158 obese pregnant women, diet intervention reduced the rate of increase in extremely large, very large, large and medium VLDL particles during gestation [48]. Whilst there was some evidence from our study that the intervention may alter GWG-metabolite relations in a few lipids, we did not identify any markers that successfully differentiated the control and intervention group directly, possibly hampered by a combination of small sample size (*N* = 50 for control/*N* = 64 for intervention) and small effect size due to the lifestyle intervention design. Over 1000 pregnant women with obesity were recruited in the UPBEAT study, but their lifestyle intervention was only marginally associated with reduced GWG amongst participants (mean difference: −0·55 kg; 95% confidence interval: −1·08 kg to −0·02 kg; *p* = 0.04) [48]. Implementation of lifestyle intervention during pregnancy is by necessity moderate since acute weight loss or excessive exercise could have adverse effects on the infant. Therefore, the consequent impact on the metabolome will be less marked than that observed after following a more severely restricted diet where alterations in protein, lipid and short chain fatty acid metabolism and potentially ketogenesis are commonly observed. Nevertheless, we see systematic alterations in the metabolome related to BMI, weight gain and WL. In order to achieve deeper insight into gestational weight gain and WL post pregnancy, future studies should account for participant advice adherence, and consider collecting more granular habitual data over the study intervention period for analysis, including changes in physical activity levels. Our work provides evidence based on the lipidomic profiles that calorie and nutrient intakes likely play a vital role in both reducing excessive GWG and in facilitating WL postpartum. Our future planned work includes utilizing the urine and lipid profiles collected in this participant sample for identifying possible diet-influenced phenotypes in the two study groups, which may in turn facilitate the monitoring of participant adherence to diet and lifestyle intervention advice.

## 4. Materials and Methods

### 4.1. Participant Recruitment

**Figure 5:**
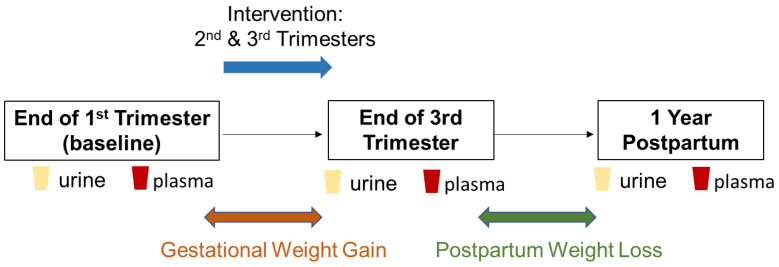
Overview of the study design workflow.

The MOMFIT Study participant recruitment and methodology are described elsewhere [14]. Briefly, ethnically diverse pregnant women aged 18–40 years with overweight or obesity (BMI 25–40 kg/m^2^) from Chicago (USA) were enrolled at 9–15 weeks of gestation in a randomized diet and lifestyle intervention trial: MOMFIT; www.clinicaltrials.gov NCT01631747. All participants signed informed consent approved by the Northwestern University Institutional Review Board. Urine and blood plasma specimens (114, including 50 Usual Care control and 64 Intervention) were collected from a subsample of participants enrolled in the trial collected at 15 weeks, 35 weeks of gestation, and at 1 year postpartum and were made available for metabolic profiling analyses (Figure 5, Table 2). Demographic information, medical and obstetric history, household food insecurity, automated self-administered 24-h dietary recall (ASA24) and sleep pattern, blood pressure measurements and maternal physical activity were assessed. Urine and blood plasma metabolite profiles were acquired using metabolic profiling. From a subset of 114 of the enrolled participants, 107 (47 Usual Care control and 60 Intervention) of these individuals were profiled whilst the remaining 7 participants were excluded due to missing or incomplete urine samples.

Both the Usual Care and Intervention participants received publicly available recommendations relevant to pregnancy including the 2008 Physical Activity Guidelines for Americans and recommendations from the American College of Obstetrics and Gynecology. The “Mama” DASH dietary intervention group was prescribed calorie goals based on height, preconception weight, physical activity level, and nutrient needs relevant for restricted total GWG (0.18–0.27 kg/week for women with overweight and obesity respectively) during the second and third trimesters. The MOMFIT intervention group participants received training in self-monitoring their diet intake using a commercially available mobile application, coaching calls from the registered dietitian nutritionist and group or webinar-based DASH diet intervention sessions [14]. They also received a simple pedometer (Accusplit accelerometer AX2710) or were encouraged to monitor activity using their smartphone tracking device and log activity in their mobile application account. The activity goal was 30 min or more of moderate walking > 10,000 steps per day.

Research has been carried out according to the international and national guidelines and regulations (including the Declaration of Helsinki), and informed consent was obtained from all subjects. Ethics approvals were obtained from the following committees: IRB office, Northwestern University (STU00053566-MOD0019); Imperial College Research Ethics Committee (18IC44500).

### 4.2. Urinary ^1^H NMR Metabolite Measurements

Urine samples were analyzed by ^1^H NMR. One-dimensional ^1^H NMR spectra were acquired on a Bruker Avance III spectrometer (Bruker BioSpin, Rheinstetten, Germany) operating at 14.1 Tesla. The spectrometer was equipped with a Bruker SampleJet system, and a 5-mm broad-band inverse configuration probe maintained at 300 K. Prior to analysis, samples were randomized and individual samples were thawed and homogenized using a vortex mixer and centrifuged at 13,000× *g* for 10 min at 4 °C to remove insoluble material. An aliquot of 540 μL of urine sample was mixed with 60 μL of a buffer solution (1.5 M KH_2_PO_4_, 2 mM NaN_3_, 1% deuterated 3-(trimethylsilyl)-[2,2,3,3-d_4_]-propionic acid sodium salt (TSP) solution, pH 7.4). Samples were transferred into an NMR tube (5 mm outer diameter Bruker SampleJet NMR tubes) and were kept at 6 °C in the Bruker SampleJet (Bruker BioSpin, Rheinstetten, Germany) unit. The ^1^H NMR spectra were acquired using a standard 1D pulse sequence using the first increment of the NOE pulse sequence to achieve suppression of the water resonance with gradients (noesygppr1d). In total, 32 transients were collected into 64 K data points using a spectral width of 12,000 Hz with a recycle delay of 4 s, a mixing time of 10 ms, and an acquisition time of 2.73 s. Line-broadening of 0.3 Hz was applied prior to Fourier transformation. All ^1^H NMR spectra were automatically phased and baseline-corrected using Topspin 3.2 software (Bruker BioSpin, Rheinstetten, Germany). The ^1^H NMR urine spectra were referenced to the TSP resonance at 0 ppm. The NMR spectra were aligned using recursive segment-wise peak alignment—an algorithm based on cross-correlation, using MATLAB 2018a (MathWorks, Natick, MA, USA). Assignment of endogenous urinary metabolites was made by reference to online compound databases, statistical methods applied to the full spectra (STOCSY [49]), and/or confirmed by 2D NMR experiments [50].

### 4.3. LC-MS Blood Lipids Measurements

Lipid profiling was performed on plasma samples using reverse-phase UPLC-MS with data acquired in positive (ESI+) and negative (ESI−) ion modes. Experiments were performed on a Waters Synapt G2-S UPLC-QTOF-MS system. Prior to analysis 400 μL of isopropanol was added to 100 μL plasma. Samples were then vortexed for 10 min at room temperature before being centrifuged at 18,000× *g* for 30 min. Approximately, 150 μL of supernatant was transferred to a glass vial with insert for analysis. A pooled study quality control sample (QC) was also generated, by mixing together a small volume from each study sample, for conditioning of the chromatographic column and to assess analytical reproducibility. A 7-point dilution series, generated with 100%, 80%, 60%, 40%, 20%, 10%, 1% of the QC sample, was used to assess linearity between metabolite concentration and signal response. An ACQUITY sample manager (Waters Corp, Milford, MA, USA) maintaining samples at 4 °C and a ACQUITY Binary Solvent Manager (Waters Corp, Milford, MA, USA) were used to inject a volume of 4 μL of extracts into an ACQUITY UPLC BEH C8 column (2.1 × 100 mm, 1.7 μm; Waters Corp). Separation was achieved with mobile phases consisting of water/isopropanol/acetonitrile 2:1:1 (5 mM ammonium acetate, 0.05% acetic acid, 20 μM phosphoric acid) as mobile phase A and isopropanol/acetonitrile 1:1 (5 mM ammonium acetate, 0.05% acetic acid) as mobile phase B over 13.25 min at a flow rate of 0.6–1 mL/min. The elution gradient was set as follow: 99–70% A (0–2 min, flow rate: 0.6 mL/min), 70–10% A (2–11.5 min, flow rate: 0.6 mL/min), 10–0.1% A (11.5–12 min, flow rate: 0.6 mL/min), 0.1–35% (12–12.55 min, flow rate: 1 mL/min), 35–70% (12.55–12.65 min, flow rate: 0.9 mL/min), 70–99% (12.65–12.75 min, flow rate: 0.8 mL/min), 99% (12.75–13.25 min, flow rate: 0.7–0.6 mL/min). The MS acquisition parameters used were as follow: capillary voltage 2 kV (both ESI+ and ESI−), cone voltage 30 V (both ESI+ and ESI−), temperature 350 °C, desolvation flow 600L/h, source temperature 100 °C. The MS data were collected at a range of 50–2000 Da with a scan time of 0.2 s and interscan delay of 0.015 s. To provide a lock-mass correction, a 400 ng/μL solution of leucine enkephalin was infused at a rate of 15 μL/min. MS spectra were acquired in continuum mode. For data preprocessing, the MS peaks were centroided using MassLynx (version 4.1, Waters Corp, Milford, MA, USA) and the data were converted to NetCDF format using DataBridge (Waters Corp, Milford, MA, USA). Chromatographic peak detection, retention time alignment, and feature grouping were carried out using XCMS (version 3.2.0) [51] in the R programming environment. Only features (*m*/*z*_RT pairs) which scale linearly according to dilution (R^2^ > 0.8 with 7-point dilution series) and showed good reproducibility (RSD < 0.3 on QC samples) were carried forward to further data analysis. Annotation of plasma lipid features was based matching of feature’s *m*/*z* against online databases such as METLIN [52], Human Metabolome Database (HMDB) [53] and LipidMaps [54], which enabled the identification of lipid class and number of carbons of the fatty acyl chains. This information was confirmed by running data-dependent (DDA) and data-independent (MS^E^) LC-MS/MS fragmentation experiments. Fatty acyl side chains compositions were determined for most features. Some features annotations are considered putative as either they lacked clear direct evidence from MS/MS collected through data dependent acquisition, or annotations were made based on ion coelution correlations to known adducts alone (Supplementary Table S1). Our analysis does not discriminate between allyl-(plasmanyl) or alkenyl (plasmenyl) linked ether lipids which differ only by the position of the C=C bond in the sn-1 position and produce the same fragment ions.

### 4.4. Statistical Analysis

Metabolite signal intensities were normalized using median fold change, and signals were log-transformed, mean-centered and scaled to unit-variance prior to conducting PCA to identify inherent structure in the spectral data relating to BMI, GWG or WL post-partum. For the initial Pearson’s correlation analyses performed against the 1127 lipid features, a false discovery rate of 0.05 was used as significance level cutoff. Linear regression analysis was performed to relate GWG and WL to plasma lipid percentage changes in the corresponding sampling periods, the plasma lipid percentage changes were calculated using the median fold change normalized data without log-transformation. Lipid % change during the 1st period (GWG) = (Lipid_W35_ − Lipid_W15_)/Lipid_W15_, and % change during the 2nd period (WL) = (Lipid_1Y_ − Lipid_W35_)/Lipid_W35_. In the follow-up linear regression analyses performed against the 57 annotated lipid species, a more stringent multiple testing correction method was applied. Bonferroni correction was applied based on the effective number of test—defined as the number of principal components required to explain 95% of variance in the LCMS metabolomics dataset, and *p*_adjusted_ value of 0.05 was used as significance level cutoff. All statistical analyses were performed using R (“The R Project for Statistical Computing” https://www.r-project.org) software environment (v3.5).

## 5. Conclusions

In conclusion, our study provides evidence that phospholipid profiles and their incorporated fatty acyl chains are possible markers of GWG and postpartum WL in metabolically healthy pregnant women with overweight/obesity. Importantly, phospholipids associated with GWG tend to also be associated with postpartum WL but as expected, in the opposite direction. Postpartum WL may be linked to the reduction in the intake of linoleic acid/conjugated linoleic acid food sources in our study sample. Alterations in blood lipids could potentially be explored to further evaluate and shed light on the biological effects and benefits of lifestyle interventions. Larger studies in independent cohorts are warranted to validate our findings.

## Figures and Tables

**Table 1:** Linear regression analysis between lipid level (fractional change), and GWG and WL. Only lipids which were found to be significantly associated with either GWG or WL are shown below (*p* threshold = 0.0017). Bonferroni correction is used to account for multiple testing.

Lipid Species	Beta GWG	95% Confidence	*p*	Beta WL	95% Confidence	*p*
PC (42:6)	−0.011	(−0.015, −0.007)	8.9 × 10^−8^	0.009	(0.001, 0.018)	3.5 × 10^−2^
SM (d17:1/24:1)	−0.011	(−0.015, −0.007)	5.6 × 10^−7^	0.013	(0.007, 0.019)	1.2 × 10^−5^
PC (O-18:1_20:4)	−0.015	(−0.022, −0.009)	2.2 × 10^−6^	0.026	(0.017, 0.036)	3.4 × 10^−7^
PC (O-20:0_20:4)	−0.018	(−0.025, −0.01)	6.2 × 10^−6^	0.006	(0.001, 0.011)	2.0 × 10^−2^
PC (40:8)	−0.013	(−0.019, −0.007)	2.4 × 10^−5^	0.011	(0.005, 0.016)	2.4 × 10^−4^
PC (16:0_20:4)	0.009	(0.005, 0.013)	2.8 × 10^−5^	−0.004	(−0.007, 0)	2.8 × 10^−2^
PC (O-18:1_16:0)	−0.011	(−0.016, −0.006)	2.9 × 10^−5^	0.010	(0.002, 0.017)	9.7 × 10^−3^
SM (d18:2/24:1)	−0.014	(−0.021, −0.008)	6.4 × 10^−5^	0.007	(0.002, 0.012)	6.4 × 10^−3^
PC (O-24:2_20:4)	−0.009	(−0.014, −0.005)	1.2 × 10^−4^	0.006	(0.002, 0.01)	5.8 × 10^−3^
PC (O-22:1_18:1)	−0.014	(−0.021, −0.007)	1.5 × 10^−4^	0.009	(0.005, 0.014)	9.8 × 10^−5^
SM (d18:1/24:1)	−0.010	(−0.016, −0.005)	4.3 × 10^−4^	0.006	(0.001, 0.011)	1.4 × 10^−2^
PC (O-16:0_22:4)	−0.012	(−0.019, −0.005)	9.0 × 10^−4^	0.003	(−0.001, 0.007)	1.6 × 10^−1^
LPC (O-18:1)	−0.014	(−0.022, −0.006)	9.7 × 10^−4^	0.047	(0.02, 0.074)	9.0 × 10^−4^
LPC (O-16:1)	−0.011	(−0.017, −0.004)	1.2 × 10^−3^	0.082	(0.034, 0.13)	9.4 × 10^−4^
PC (O-24:1_20:4)	−0.008	(−0.013, −0.003)	1.2 × 10^−3^	0.007	(0.003, 0.012)	2.7 × 10^−3^
PI (16:0_16:1)	0.140	(0.055, 0.226)	1.5 × 10^−3^	−0.016	(−0.026, −0.006)	1.3 × 10^−3^
PC (16:0_22:5)	0.022	(0.009, 0.036)	1.6 × 10^−3^	−0.008	(−0.014, −0.003)	3.8 × 10^−3^
SM (d18:2/14:0)	0.009	(0.002, 0.016)	1.2 × 10^−2^	−0.013	(−0.019, −0.007)	1.1 × 10^−4^
LPC (O-16:0)	−0.009	(−0.016, −0.002)	1.4 × 10^−2^	0.064	(0.03, 0.098)	3.3 × 10^−4^
PC (18:1_18:2)	0.079	(0.013, 0.145)	2.0 × 10^−2^	−0.014	(−0.022, −0.006)	7.1 × 10^−4^
PC (16:1_18:2)	0.013	(0.002, 0.025)	2.2 × 10^−2^	−0.012	(−0.017, −0.006)	1.6 × 10^−4^
PI (16:0_18:2)	0.012	(−0.004, 0.029)	1.4 × 10^−1^	−0.009	(−0.014, −0.004)	8.9 × 10^−4^
LPS(O-18:0)	−0.005	(−0.012, 0.002)	1.8 × 10^−1^	0.156	(0.082, 0.229)	5.3 × 10^−5^
DG (18:1_18:2)	−0.013	(−0.041, 0.015)	3.4 × 10^−1^	−0.010	(−0.017, −0.004)	1.5 × 10^−3^
PC (14:0_18:2)	0.007	(−0.017, 0.031)	5.7 × 10^−1^	−0.017	(−0.025, −0.01)	1.3 × 10^−5^
PC (15:0_18:2)	0.000	(−0.009, 0.009)	9.6 × 10^−1^	−0.008	(−0.012, −0.003)	9.8 × 10^−4^

**Table 2:** Characteristics of study volunteers. Summary statistics of the subsample of the MOMFIT trial (114 individuals: 50 control and 64 intervention) included in the metabolic profiling study. Values represent means and standard errors, and *p*-values (calculated through 2-tailed Student’s *t* test).

Characteristics	Control (*n* = 50)	Intervention (*n* = 64)	*p* Values
Age (y, 15 weeks)	34 ± 4	34 ± 4	0.16
Weight (kg, 35 weeks)	95 ± 12	91 ± 13	0.07
BMI (kg/m^2^, 35 weeks)	35 ± 4	34 ± 4	0.06
Gestational Weight Gain (kg)	10.6 ± 5.1	9.6 ± 4.6	0.14
Weight Loss postpartum (kg)	9.1 ± 7.2	9.4 ± 6.7	0.43

## Supplementary Materials

The following are available online at https://www.mdpi.com/2218-1989/10/12/498/s1, Figure S1: Principal Component Analysis of NMR urine samples, Figure S2: Principal Component Analysis of LC-MS plasma samples, Table S1: Details of Lipid annotations.
