# Supplementary material for: Metabolic Signatures of Gestational Weight Gain and Postpartum Weight Loss in a Lifestyle Intervention Study of Overweight and Obese Women
Source: Metabolites. 2020 Dec 4;10(12):498. doi: 10.3390/metabo10120498 (PMC7761920; doi:10.3390/metabo10120498)
Supplement: Supplementary file 1 [file metabolites-10-00498-s001.pdf]

Article

Metabolic Signatures of Gestational Weight Gain and Postpartum Weight Loss in a Lifestyle Intervention Study of Overweight and Obese Women

Chung-Ho E. Lau [1,2,*], Victoria Taylor-Bateman [1,3], Panagiotis A. Vorkas [4,5], Gonçalo Graça [6], Thanh-Huyen T. Vu [7], Lifang Hou [7], Elena Chekmeneva [8], Timothy M. D. Ebbels [6], Queenie Chan [2,9], Linda Van Horn [7,†] and Elaine Holmes [1,10,*,†]

[1] Section of Nutrition, Department of Metabolism, Digestion and Reproduction, Imperial College London, London, SW7 2AZ, UK;

[2] Department of Epidemiology and Biostatistics, School of Public Health, Imperial College London, London, W2 1PG, UK; q.chan@imperial.ac.uk

[3] William Harvey Research Institute, Barts and The London School of Medicine and Dentistry, Queen Mary University of London, London, EC1M 6BQ, UK; v.j.taylor-bateman@qmul.ac.uk

[4] Section of Biomolecular Medicine, Department of Metabolism, Digestion and Reproduction, Imperial College London, London, SW7 2AZ, UK; p.vorkas@imperial.ac.uk

[5] Institute of Applied Biosciences, Centre for Research and Technology Hellas, 57001 Thessaloniki, Greece

[6] Section of Bioinformatics, Department of Metabolism, Digestion and Reproduction, Imperial College London, London, SW7 2AZ, UK; g.gomes-da-graca@imperial.ac.uk (G.G.); t.ebbels@imperial.ac.uk (T.M.D.E.)

[7] Department of Preventive Medicine, Feinberg School of Medicine, Northwestern University, Chicago, IL, 60611, USA; huyenvu@northwestern.edu (T.-H.T.V.); l-hou@northwestern.edu (L.H.); lvanhorn@northwestern.edu (L.V.H.)

[8] National Phenome Centre and Section of Bioanalytical Chemistry, Department of Metabolism, Digestion and Reproduction, Imperial College London, Hammersmith Campus, IRDB Building, London, W12 0NN, UK

[9] MRC Centre for Environment and Health, Imperial College London, London, W2 1PG, UK

[10] Centre for Computational and Systems Medicine, Health Futures Institute, Murdoch University, Perth, WA, 6150, Australia

* Correspondence: chungho.lau@imperial.ac.uk (C.-H.E.L.); elaine.holmes@murdoch.edu.au (E.H.)

† Joint Senior Authors.

Received: 2 November 2020; Accepted: 1 December 2020; Published: date

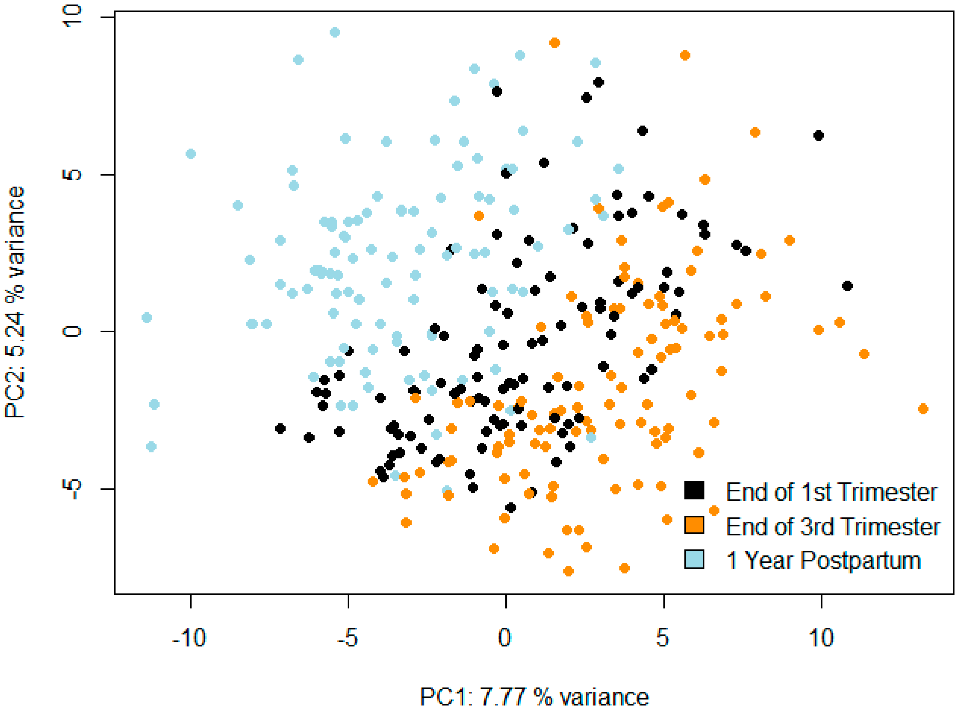

Supplementary Figure S1 Principal Component Analysis of NMR urine samples.

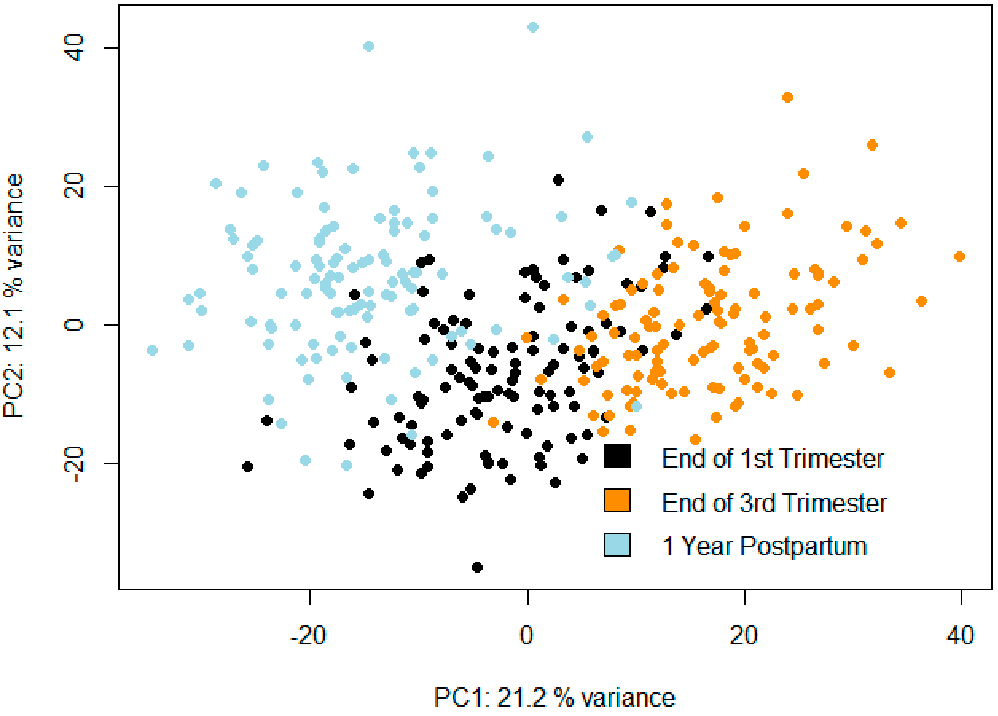

Supplementary Figure S2 Principal Component Analysis of LC-MS plasma samples.

Supplementary Table S1 Details of Lipid annotations.

Lipid	RT(min)	m/z	Annotation	Confidence of Annotation
LPS(O-18:0)	1.64	512.34	[M+H]	Match by accurate mass
LPC(22:6)	1.73	568.34	[M+H]	MS/MS (DDA)
LPC(20:4)	1.80	545.34	[M+1+H]	MS/MS (DDA)
LPC(18:2)	1.80	520.34	[M+H]	MS/MS (DDA)
LPE(16:0)	2.08	454.29	[M+H]	MS/MS (DDA)
LPC(18:1)	2.18	522.36	[M+H]	MS/MS (DDA)
LPC(O-16:0)	2.27	483.36	[M+H]	MS/MS (DDA)
LPC(O-16:1)	2.27	481.35	[M+H]	MS/MS (DDA)
LPC(17:0)	2.30	510.36	[M+H]	MS/MS (DDA)
LPC(O-18:1)	2.41	508.38	[M+H]	MS/MS (DDA)
LPC(O-18:0)	2.88	510.39	[M+H]	MS/MS (DDA)
SM(d30:1)	4.56	647.51	[M+H]	MS/MS (DDA)
SM(d18:2/14:0)	4.69	674.53	[M+1+H]	MS/MS (DDA)
PI(16:0_16:1)	5.05	549.49	[M+H-260]	MS/MS (DDA)
PI(16:0_18:2)	5.21	857.51	M+Na]	MS/MS (DDA)
PI(16:0_20:4)	5.22	859.53	[M+H]	MS/MS (DDA)
SM(d18:2/16:0)	5.45	723.54	M+Na]	MS/MS (DDA)
PC(30:1)	5.50	705.53	[M+H]	MS/MS (DDA)
PI(32:0)	5.57	551.50	[M+H-260]	MS/MS (DDA)
PC(14:0_18:2)	5.67	733.55	[M+H+3]	MS/MS (DDA)
SM(d17:1/16:0)	5.67	416.36	Unassigned adduct	Match by ion coelution
PI(34:1)	5.74	577.52	[M+H-260]	MS/MS (DDA)
PC(40:8)	5.76	1228.76	Unassigned adduct	Match by ion coelution
PC(16:1_18:2)	5.88	758.56	[M+2+H]	MS/MS (DDA)
PI(18:0_20:2)	5.91	863.56	[M+H]	MS/MS (DDA)
PC(15:0_18:2)	6.02	745.56	[M+1+H]	MS/MS (DDA)
SM(d18:1/16:0)	6.04	763.63	[M+CH3CN+NH4]	MS/MS (DDA)
PC(42:6)	6.07	844.61	[M+H-H2O]	MS/MS (DDA)
PC(16:0_16:1)	6.22	733.56	[M+1+H]	MS/MS (DDA)
PC(16:0_22:5)	6.33	1491.12	Unassigned adduct	Match by ion coelution
PC(16:0_20:4)	6.35	1945.41	Unassigned adduct	Match by ion coelution
PC(17:0_22:6)	6.50	820.58	[M+H]	MS/MS (DDA)
PE(34:2)	6.50	716.52	[M+H]	MS/MS (DDA)
PC(18:1_18:2)	6.58	1960.95	Unassigned adduct	Match by ion coelution
PC(16:0_22:4)	6.81	596.57	Unassigned adduct	Match by ion coelution
PC(O-18:1_20:4)	6.88	795.61	[M+H]	MS/MS (DDA)
PC(16:0_18:1)	6.91	820.63	Unassigned adduct	Match by ion coelution
PC(18:0_18:2)	7.08	786.75	[M+H]	MS/MS (DDA)
PC(O-18:1_20:3)	7.22	818.60	M+Na]	MS/MS (DDA)
PC(15:0_20:1)	7.25	774.60	[M+H]	Match by accurate mass
PC(O-16:0_22:4)	7.25	796.62	[M+H]	Match by accurate mass
PC(18:0_20:3)	7.28	813.62	[M+H]	MS/MS (DDA)
PC(O-18:1_16:0)	7.33	746.60	[M+H]	Match by accurate mass
PE(O-20:0_22:6)	7.33	776.56	[M+H]	MS/MS (DDA)
PC(O-18:1_18:2)	7.45	770.60	[M+H]	MS/MS (DDA)
DG(18:1_18:2)	7.61	601.52	[M+H-H2O]	MS/MS (DDA)
SM(d18:2/24:1)	7.66	811.67	[M+H]	MS/MS (DDA)
PC(18:0_22:4)	7.76	838.63	[M+H]	MS/MS (DDA)
SM(d17:1/24:1)	7.89	831.69	Unassigned adduct	Match by ion coelution
DG(34:1)	7.99	617.51	[M+Na]	MS/MS (DDA)
DG(36:2)	8.09	603.53	[M+H-H2O]	MS/MS (DDA)
PC(O-22:1_18:1)	8.14	851.67	M+1+Na]	Match by accurate mass
SM(d18:1/24:1)	8.16	933.64	[M+H2PO4+Na+H]	MS/MS (DDA)
PC(O-20:0_20:4)	8.16	826.66	[M+H]	MS/MS (DDA)
PC(O-24:2_20:4)	8.24	876.68	[M+H]	Match by accurate mass
PC(O-24:1_20:4)	8.72	879.70	[M+1+H]	MS/MS (DDA)
HexCer(d18:1/24:0)	8.91	812.70	[M+H]	MS/MS (DDA)
